# Supplementary material for: Abrupt and spontaneous strategy switches emerge in simple regularised neural networks
Source: PLoS Comput Biol. 2024 Oct 21;20(10):e1012505. doi: 10.1371/journal.pcbi.1012505 (PMC11527165; doi:10.1371/journal.pcbi.1012505)
Supplement: S1 Text — Supplementary information file including additional analyses on behavioural effects without feedback and late onset of low coherence trials, effects of regularisation type on network behaviour, the hidden layer model, weight and gate differences between L1- and L2-regularised networks as well as Gaussian noise differences at weights and gates between insight and no-insight networks. This file includes Fig A (Switch-aligned performance and switch point distributions for L1- and L2-regularised neural networks with a 48 unit hidden layer each), Fig B (Illustrations of models and respective parameters), Fig C (Performance on highest motion noise trials and model predictions for every human participant), Fig D (Performance on highest motion noise trials and model predictions for every network), Fig E (Switch-aligned performance for insight group and no-insight group), Fig F (Switch-aligned performance and overlap between classification and self-reported colour use), Fig G (Trial-wise insight-like strategy improvements for 5% motion coherence trials), Fig H (L2 networks: Task performance and insight-like strategy switches) and Fig J (Comparison of gate weight magnitude and influence of λ on insight-like behaviour across L1- and L2-regularised networks). Figure legends see inside S1 Text. (PDF) [file pcbi.1012505.s001.pdf]

Supplementary Material: Abrupt and spontaneous strategy switches emerge in simple regularised neural networks

Anika T. Löwe[1,2,3]*, Léo Touzo[4], Paul S. Muhle-Karbe[5,6,7], Andrew M. Saxe[8,9,10], Christopher Summerfield[5]¶, Nicolas W. Schuck[3,1,2]¶

1 Max Planck Research Group NeuroCode, Max Planck Institute for Human Development, Berlin, Germany
2 Max Planck UCL Centre for Computational Psychiatry and Ageing Research, Berlin, Germany
3 Institute of Psychology, Universität Hamburg, Hamburg, Germany
4 Laboratoire de Physique de l'Ecole Normale Supérieure, CNRS, ENS, Université PSL, Sorbonne Université, Université Paris Cité, Paris, France
5 Department of Experimental Psychology, University of Oxford, Oxford, UK
6 School of Psychology, University of Birmingham, Birmingham, UK
7 Centre for Human Brain Health, University of Birmingham, Birmingham, UK
8 Gatsby Computational Neuroscience Unit, University College London, London, UK
9 Sainsbury Wellcome Centre, University College London, London, UK
10 CIFAR Azrieli Global Scholar, CIFAR, Toronto, Canada

*corresponding author: loewe@mpib-berlin.mpg.de
¶ Senior author

1 Effects of Feedback and Low Coherence

Behaviour Without Feedback

To exclude the possibility that insight-like behavioural switches were driven by the feedback signal acting as an external reward cue after the contingency change, we ran another task version without trial-wise feedback. In this experiment (N = 61), participants were presented with the same random dot motion stimuli, but motion coherence ranged from 0% to 50% in increments of 10. 0% coherence trials were thus truly random and could only be solved if participants had an insight about the predictiveness of the colour. Instead of learning feature relevance from trial-wise feedback, participants were here instructed about the stimulus-response mapping between the different dot motion directions and respective response keys and performed three training blocks (out of which the first two administered trial-wise feedback) before they started with the main task. During the main experiment, the only feedback participants received was their average accuracy at the end of each block. The onset of the *motion and colour phase* occurred after two task blocks of the main experiment.

We then used the same procedure of classifying insight-like behaviour by assessing in how many participants the steepness of the performance increase exceeded the chance level defined by the baseline distribution of steepness. About a fifth of participants (6/29, 20.1%) had steepness values larger than the 100% percentile of the control distribution. As expected, insight subjects also started to perform significantly better in 0% coherence trials once the *motion and colour phase* started, (mean proportion correct in *motion and colour phase*: $M = 75 \pm 6\%$), compared to participants without insight ($M = 56 \pm 5\%$) ($t(18.4) = 4.8$, $p < .001$, $d = 1.4$).

Late Onset of Difficult Trials

To further control whether the most difficult trials with the lowest motion coherence were driving insight-like behaviour, we ran another task version (N=29) of the same structure as described above, but delayed the onset of the two lowest motion coherence levels, 5% and 10%, until the sixth task block. In this task version subjects were also instructed about the motion relevance and received two training blocks, but were also presented with trial-wise feedback during the main experiment. There was no performance difference on 5% coherence trials in the last two task blocks between this and another task version with low coherence trials from the first block on ($M = 89 \pm 3\%$ vs $M = 89 \pm 3\%$, $t(54.1) = 0.04$, $p = 0.97$, $d = 0.01$), demonstrating that the majority of subjects had switched to using colour irrespective of difficult trials. These results are in line with earlier work by Gaschler et al. [21] using a similar version of our task.

2 Effects of Regularisation Type on Network Behaviour

Following our observation that L1-regularised networks exhibited human-like insight behaviour, we investigated whether this was specific to the form of regularisation. We therefore first trained otherwise identical networks with a L2-regularisation term on the gate weights. We hypothesised that L2-regularisation would also lead to competitiveness between input nodes, but to a lower extent than L1-regularisation. We reasoned that in particular the fact that during the *motion phase* the networks motion weights would not shrink as close to 0 would lead to more frequent and earlier insight-like behavioural switches.

While L2-regularised gate weights indeed led to switches that were similar to those previously observed in their abruptness (Fig HC in S1 Text), as predicted such insight-like behaviours were much more frequent and clustered: 96% of networks switched to a colour strategy, with a switch point distribution that was much more centred around the onset of the colour predictiveness (Fig HF in S1 Text, average delay of 2 task blocks ($SD = 1.1$) corresponding to 100 trials after onset of the colour correlation (*motion and colour phase*). This was significantly shorter than for L1-regularised networks ($M = 1.05 \pm 1.1$ vs. $M = 1.75 \pm 1.05$, $t(59.6) = 4$, $p < 0.001$, $d = 0.9$) and also differed from a uniform distribution taking into account the hazard rate (Exact two-sided Kolmogorov-Smirnov test: $D(95) = 0.26$, $p = 0.005$). Additionally, performance on the lowest coherence level in the last block of the *colour and motion phase* before colour instruction was centred just below ceiling and thus did not indicate a range of colour use like humans and L1-regularised networks ($M(L2 - networks) = 97\% \pm 2\%$ vs. $M(humans) = 82 \pm 17\%$, $t(101.6) = -8.8$, $p < .001$, $d = 1.25$, Fig IC in S1 Text). While L2-regularised networks thus showed abrupt behavioural transitions, they failed to show the other two key characteristics of insight: selectivity and delay.

Next, we investigated network behaviour when no regularisation was applied. In non-regularised networks, the effects observed in L2-regularised networks are enhanced. 99% of the networks started using colour inputs (Fig JA in S1 Text), but colour use occurred in a more linear, less abrupt way than for L1- or L2-regularised networks. Additionally, there was very little delay of only 1.4 task blocks (70 trials, (± 0.25)) between onset of the *motion and colour phase* and the start of the networks making use of the colour input predictiveness (Fig JB in S1 Text). As for L2-networks, this delay was significantly shorter than for L1-regularised networks ($M = 0.7 \pm 0.55$ vs. $M = 1.75 \pm 1.05$, $t(49.3) = 6.6$, $p < 0.001$, $d = 1.6$) and also differed from a uniform distribution taking into account the hazard rate (Exact two-sided Kolmogorov-Smirnov test: $D(98) = 0.35$, $p < .001$). Similarly, performance on the lowest coherence level in the last block indicated that all networks used colour inputs ($M = 100\% \pm 0.3\%$ vs. $M = 82 \pm 17\%$, $t(98) = -10.4$, $p < .001$, $d = 1.5$, Fig JC in S1 Text). Thus non-regularised networks also did not show the insight key behavioural characteristics of selectivity and delay.

3 Hidden Layer Model

In order to verify that our results were not merely an artefact of the oversimplified models we used, we tested the task on a simple deep neural network that had one additional hidden layer of fully connected linear units.

The linear neural network received two inputs, x_m and x_c, corresponding to the stimulus motion and colour, respectively, and had two output nodes, $\hat{y}$, as well as one hidden layer of 48 units. Importantly, each weight connecting the inputs with a hidden unit had one associated multiplicative gate g. To introduce competitive dynamics between the input channels, we again applied L1-regularisation on the gate weights g.

The network was trained on the Cross Entropy loss using stochastic gradient descent with $\lambda = 0.002$ and $\alpha = 0.1$.

As for the one-layer network, we trained this network on a curriculum precisely matched to the human task, and adjusted hyperparameters (noise levels), such that baseline network performance and learning speed were carefully equated between humans and networks (see Methods).

We employed the same analysis approach to detect insight-like behaviour (see Methods for details) by running simulations of a "control" network of the same architecture, but without correlated features and therefore without colour predictiveness in the *motion and colour phase.* We found that

when we applied L1-regularisation with a regularisation parameter of $\lambda = 0.002$ on the gate weights, 18.2% of the networks exhibited *abrupt* and *delayed* learning dynamics, resembling insight-like behaviour in humans (Fig AA in S1 Text) and thereby replicating the key insight characteristics suddenness and selectivity. Insight-like switches to the colour strategy thereby again improved the networks' performance significantly. Using the same parameters, experimental setup and analyses, but applying L2-regularisation on the gate weights g, yielded an insight-like switch rate of 51.5% (Fig AB in S1 Text).

We again also observed a wider distribution of delays, the time point when the switches in the *motion and colour phase* occurred in insight networks, for L1-regularised networks with a hidden layer (Fig AC and Fig AD in S1 Text).

Taken together, these results mirror our observations from network simulations with a simplified setup. We can thereby confirm that our results of L1-regularised neural networks' behaviour exhibiting all key characteristics of human insight behaviour (suddenness, selectivity and delay) are not an artefact of the one-layer linearity.

4 Weight and Gate Differences between L1- and L2-regularised Networks

At correlation onset (first trial of *motion and colour phase*), neither motion nor colour weights differed (motion: $M = 3.5 \pm 0.6$ vs $M = 3.4 \pm 0.5$, $t(192.7) = 1.2$, $p = 0.2$, $d = 0.2$, colour: $M = 0.8 \pm 0.6$ vs $M = 0.8 \pm 0.5$, $t(189.2) = 0.4$, $p = 0.7$, $d = 0.1$). After learning, however, i.e. at the last trial of the *motion and colour phase*, the average absolute size of the colour weights was higher in L2- compared to L1-networks ($M = 2.6 \pm 2.2$ vs $M = 4.7 \pm 0.7$, $t(115.1) = -9$, $p < .001$, $d = 1.3$), while the reverse was true for motion weights ($M = 3.4 \pm 0.7$ vs $M = 2.8 \pm 0.6$, $t(194.9) = 5.6$, $p < .001$, $d = 0.8$). For gate weights, differences between L1- and L2-networks are already apparent at correlation onset (first trial of *motion and colour phase*), where the mean of the motion gate was 0.53 for L1-networks and 0.58 for L2-networks, and hence lower in L1 networks, albeit not significantly ($t(195.1) = -1$, $p = 0.3$, $d = 0.1$, see Fig DE in S1 Text). In addition, the average absolute size of the colour gate weights was higher in L2- compared to L1-networks ($M = 0.04 \pm 0.05$ vs $M = 0.002 \pm 0.006$, respectively, $t(100.6) = -7.2$, $p < 0.001$, $d = 1$). The respective distributions also reflected these effects. L1-networks had a much more narrow distribution for colour gates and just slightly narrower distribution for motion gates (L1: colour gates: 0 to 0.04, motion gates: 0 to 1.3, L2: colour gates: 0 to 0.2, motion gates: 0 to 1.4) After learning, i.e. at the last trial of the *motion and colour phase*, the mean colour gate size still was lower in L1- compared to L2-regularised networks ($M = 0.4 \pm 0.4$ vs $M = 0.8 \pm 0.2$, $t(169.1) = -9.3$, $p < 0.001$, $d = 1.3$), while the reverse was true for motion gates ($M = 0.3 \pm 0.3$ vs $M = 0.2 \pm 0.2$, $t(152.4) = 3.9$, $p < 0.001$, $d = 0.6$, see Fig DF in S1 Text). This was again also reflected in the respective distributions with L1-networks having much wider distributions for motion and slightly shorter width for colour gates (L1: colour gates: 0 to 1.2, motion gates: 0 to 1.3, L2: colour gates: 0 to 1.3, motion gates: 0 to 0.7).

5 Gaussian Noise Differences at Weights and Gates between Insight and No-Insight Networks

Comparing Gaussian noise $\xi \sim \mathcal{N}(0, \sigma_\xi^2)$ at the weights and gates around the individually fitted switch points revealed no differences between insight and no-insight networks for either motion or colour weights (colour weights: $M = -0.08 \pm 1$ vs. $M = 0.04 \pm 0.8$; $t(89.5) = -0.6$, $p = 0.5$, motion weights: $M = 0.5 \pm 0.3$ vs. $M = 0.6 \pm 0.3$; $t(93.1) = -1.7$, $p = 0.09$) or gates (colour gates: $M = -0.1 \pm 0.9$ vs. $M = 0.1 \pm 0.9$; $t(95.3) = 0.8$, $p = 0.44$, motion gates: $M = 0.2 \pm 0.6$ vs. $M = -0.3 \pm 0.8$; $t(94.4) = 2$, $p = 0.05$). There also were no σ_ξ differences at either the start of learning (first trial of the *motion and colour phase*) (colour weights: $M = -0.06 \pm 0.8$ vs. $M = -0.03 \pm 0.5$; $t(78.1) = -0.2$, $p = 0.8$, motion weights: $M = 0.08 \pm 0.7$ vs. $M = 0.07 \pm 0.7$; $t(96.7) = 1$, $p = 0.3$, colour gates: $M = 0 \pm 0.6$ vs. $M = -0.2 \pm 0.7$; $t(97) = 1.6$, $p = 0.1$, motion gates: $M = -0.04 \pm 0.6$ vs. $M = -0.07 \pm 0.7$; $t(97) = 0.2$, $p = 0.8$) or end of learning (last trial of the *motion and colour phase*)(colour weights: $M = 0.05 \pm 1.3$ vs. $M = 0.08 \pm 1.1$; $t(92.7) = -0.1$, $p = 0.9$, motion weights: $M = 0 \pm 1.2$ vs. $M = -0.02 \pm 1.1$; $t(95.6) = 0.04$, $p = 1$, colour gates: $M = 0.2 \pm 1.1$ vs. $M = -0.2 \pm 1.2$; $t(97) = 1.7$, $p = 0.09$, motion gates: $M = -0.1 \pm 1.3$ vs. $M = 0.05 \pm 1.3$; $t(96) = -0.7$, $p = 0.5$).

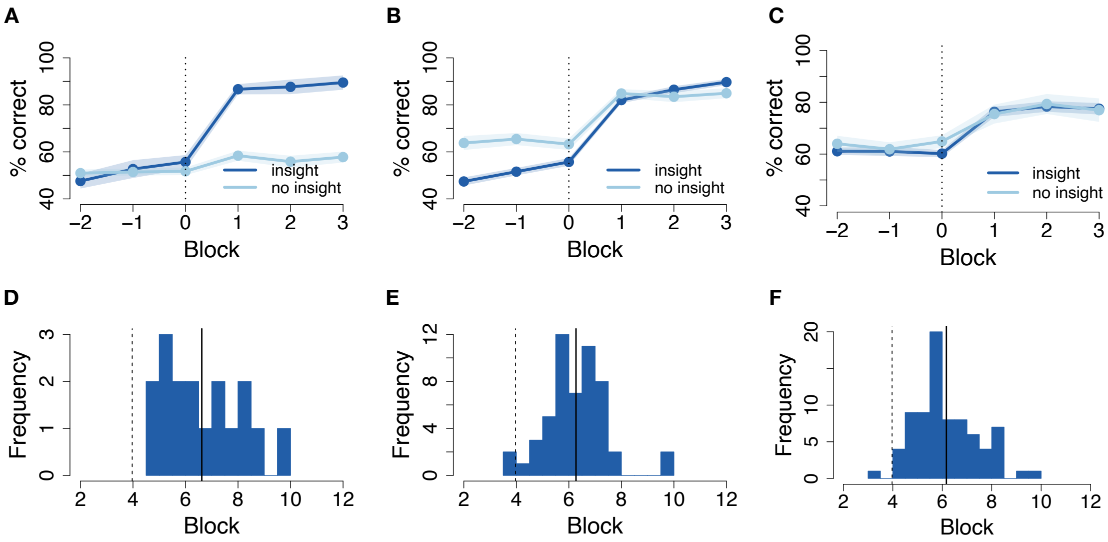

Fig A: Switch-aligned performance and switch point distributions for L1- and L2-regularised neural networks with a 48 unit hidden layer each. Blocks shown are halved task blocks (50 trials each). Error shadows signify SEM. **(A)** Switch-aligned performance for insight (18/99) and no-insight groups (81/99) respectively for L1-regularised networks with a hidden layer. **(B)** Switch-aligned performance for insight (51/99) and no-insight (48/99) L2-regularised neural networks with a hidden layer. **(C)** Switch-aligned performance for insight (78/99) and no-insight (21/99) non-regularised neural networks with a hidden layer. **(D)** Switch point distributions for L1-regularised insight networks with a hidden layer. **(E)** Switch point distributions for L2-regularised insight neural networks. Dashed vertical line marks onset of colour predictiveness. **(F)** Switch point distributions for non-regularised insight neural networks. Dashed vertical line marks onset of colour predictiveness.

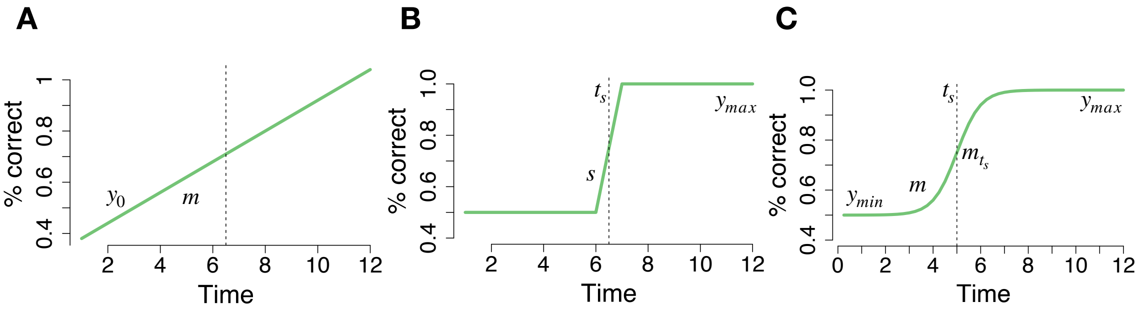

Fig B: Illustrations of models and respective parameters. **(A)** Linear function with free parameters intercept y_0 and slope m. **(B)** Step function with free parameters inflection point t_s and function maximum y_{max}. **(B)** Generalised logistic regression function with free parameters slope m, inflection point t_s and function maximum y_{max}.

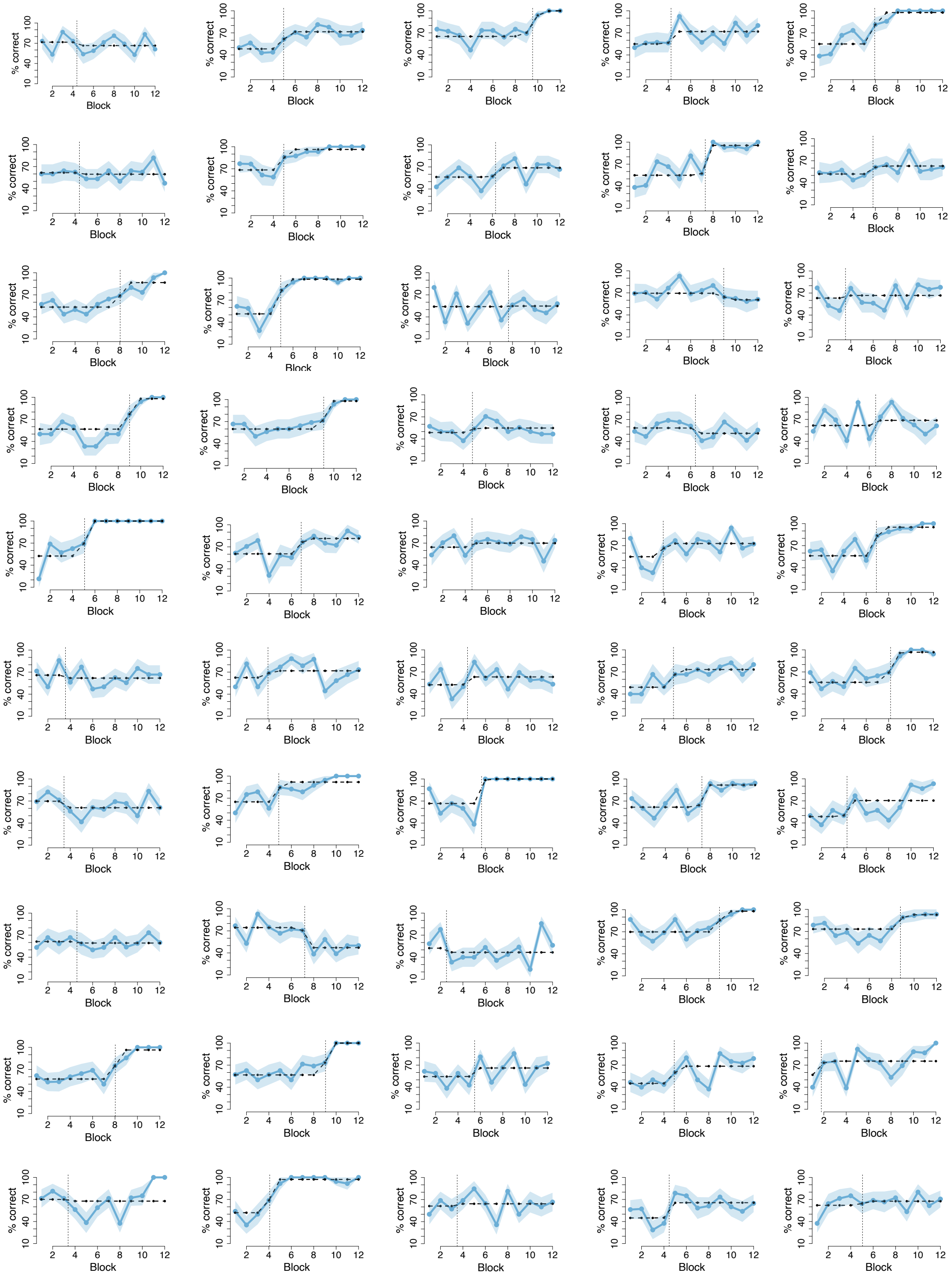
% correct
10 40 70 100
Block
2 4 6 8 10 12

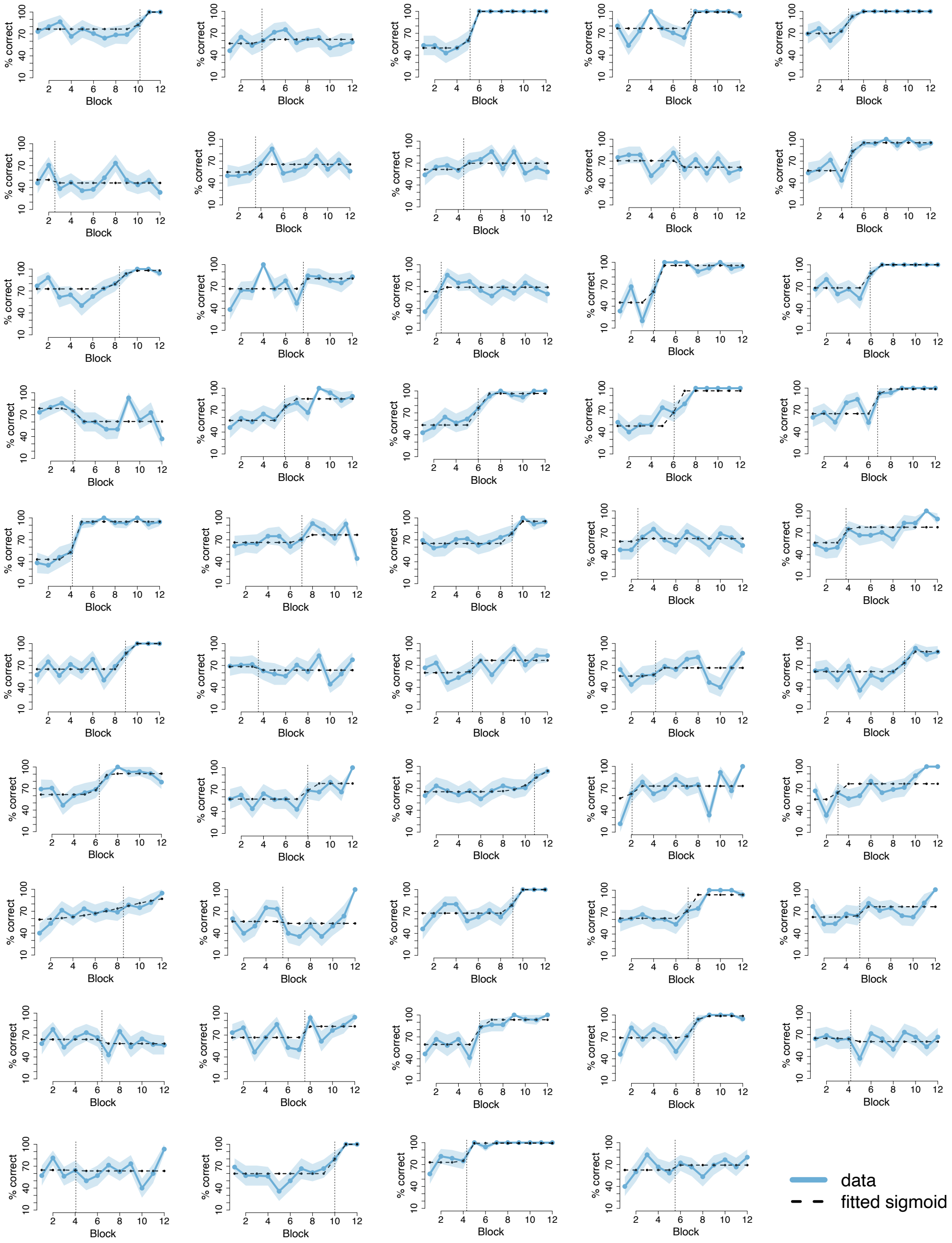

Fig C: Performance on highest motion noise trials (blue) and model predictions (black) for every human participant. Blocks shown are halved task blocks (50 trials each). Error shadows signify SEM.

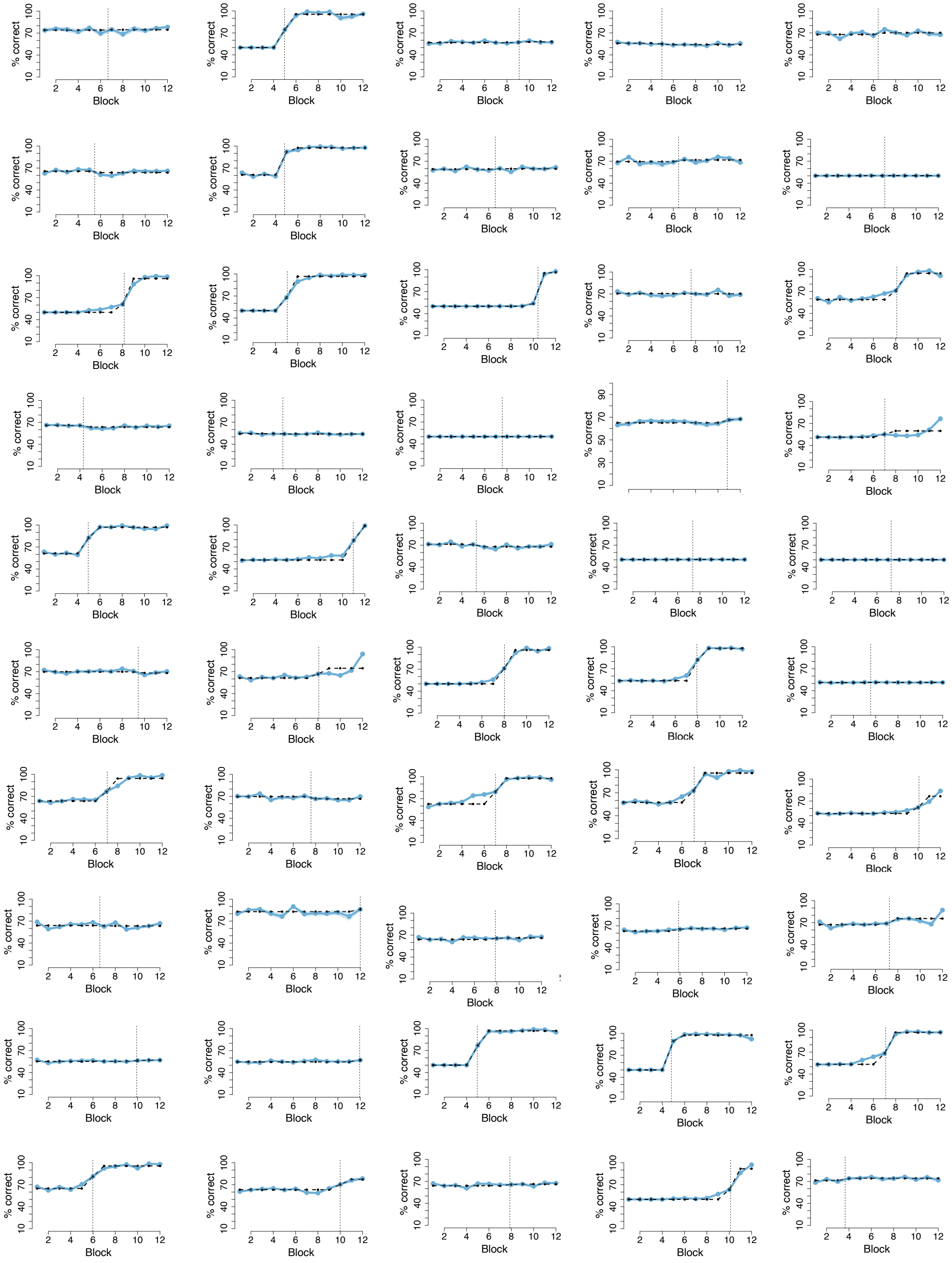
% correct
Block

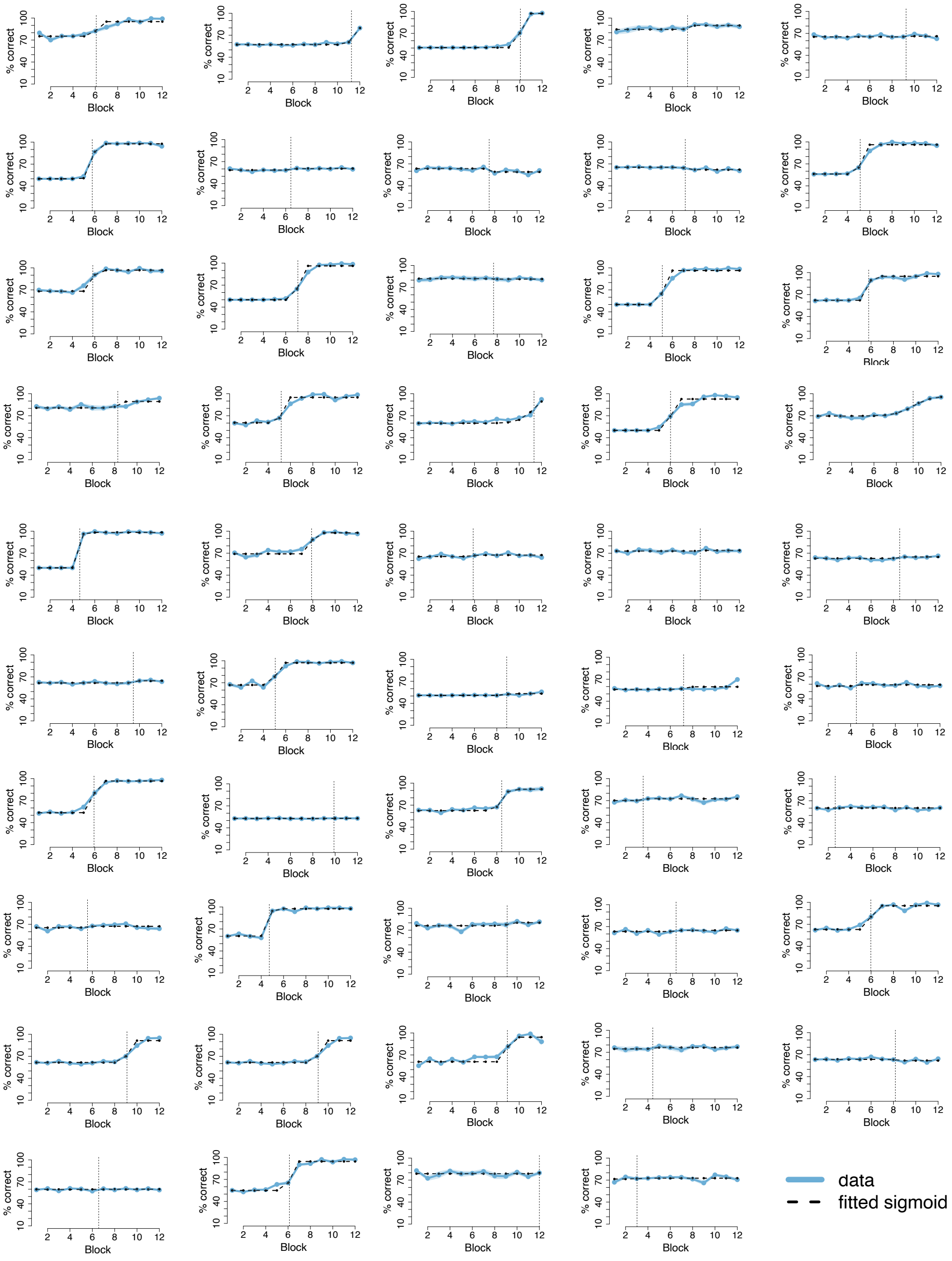

Fig D: Performance on Performance on highest motion noise trials (blue) and model predictions (black) for every L1-regularised neural network. Blocks shown are halved task blocks (50 trials each). Error shadows signify SEM.

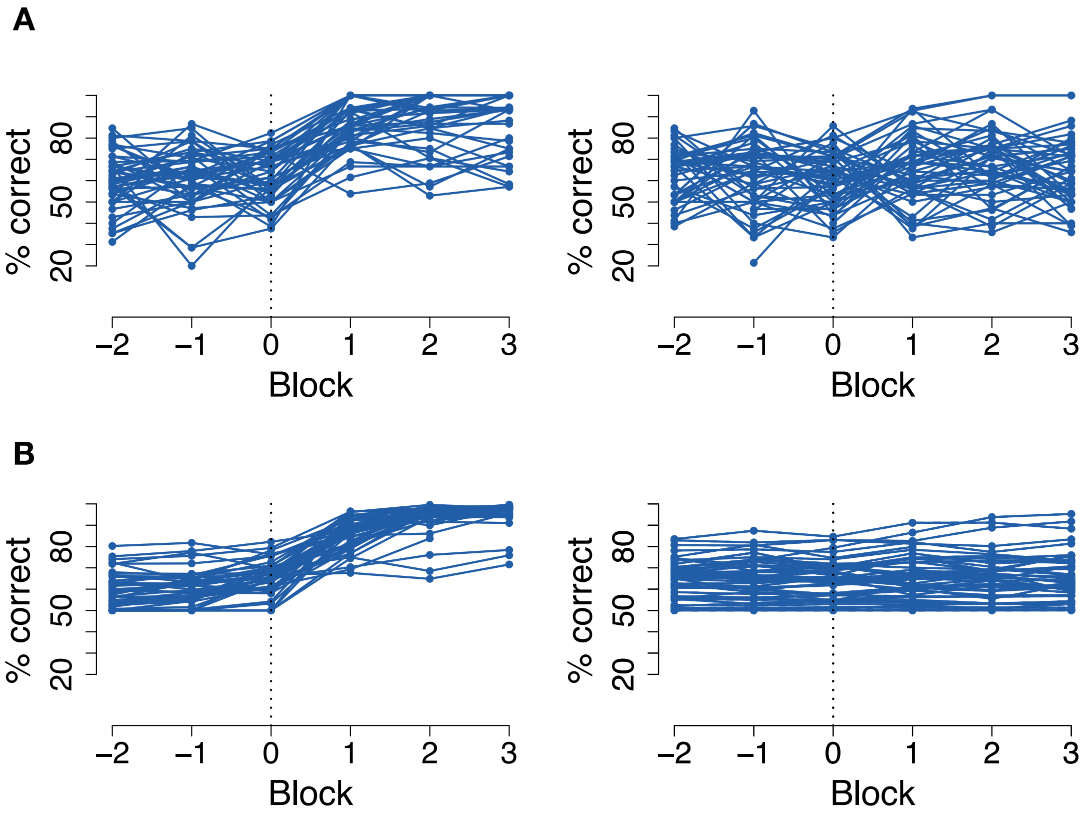

Fig E: Switch-aligned performance for insight group (left) and no-insight group (right). **(A)** Human insight group (49/99). **(B)** L1-regularised neural network insight group (48/99).

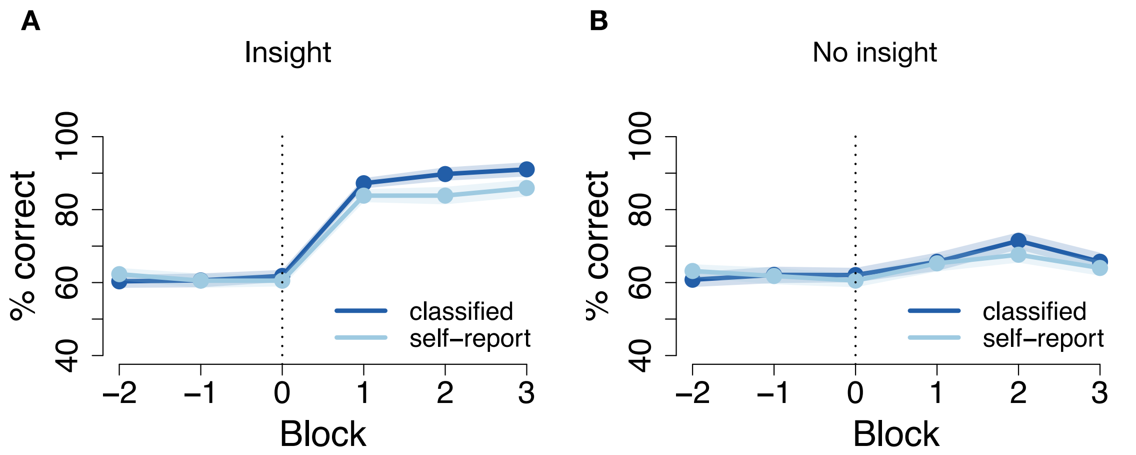

Fig F: Switch-aligned performance and overlap between classification and self-reported colour use. **(A)** Switch-aligned performance and overlap (39) between classified insight subjects (49/99) and self-reported colour use (57/99). **(B)** Switch-aligned performance and overlap (32) between classified no-insight subjects (50/99) and self-reported no colour use (42/99).

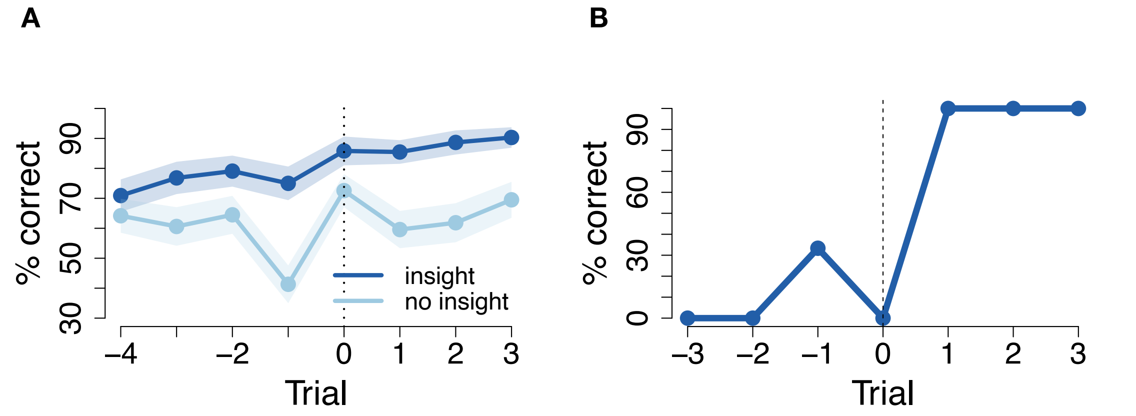

Fig G: Trial-wise insight-like strategy improvements for 5% motion coherence trials **(A)** Trial-wise switch-aligned performance between classified insight subjects (48/99) and no insight subjects (51/99). **(B)** Trial-wise switch-aligned performance for an example subject.

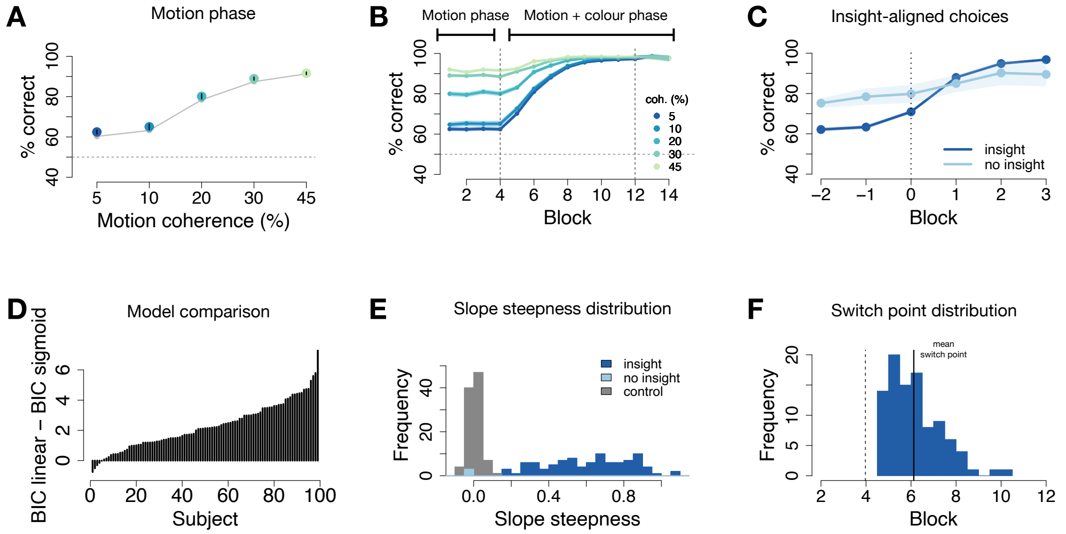

Fig H: L2 networks: Task performance and insight-like strategy switches **(A)** Accuracy (% correct) during the *motion phase* increases with increasing motion coherence. Blocks shown are halved task blocks (50 trials each). N = 99, error bars signify SEM. Grey line is human data for comparison. **(B)** Accuracy (% correct) over the course of the experiment for all motion coherence levels. First dashed vertical line marks the onset of the colour predictiveness (*motion and colour phase*), second dashed vertical line the "instruction" about colour predictiveness. N = 99, error shadows signify SEM. **(C)** Switch point-aligned accuracy on lowest motion coherence level for insight (95/99) and no-insight (4/99) networks. Blocks shown are halved task blocks (50 trials each). Error shadow signifies SEM. **(D)** Difference between BICs of the linear and sigmoid function for each network. **(E)** Distributions of fitted slope steepness at inflection point parameter for control networks and classified insight and no-insight groups. **(F)** Distribution of switch points for insight networks. Dashed vertical line marks onset of colour predictiveness. Blocks shown are halved task blocks (50 trials each).

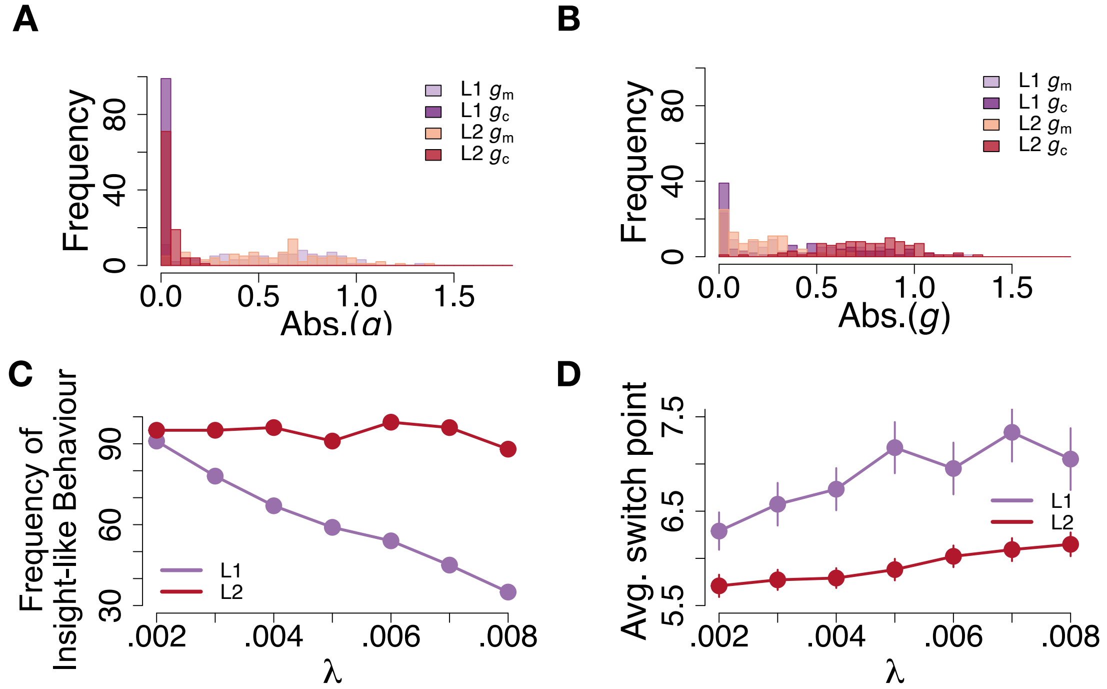

Fig I: Comparison of gate weight magnitude and influence of λ on insight-like behaviour across L1- and L2-regularised networks. Gate weight magnitude for colour and motion gate weights at the first trial **(A)** and the last trial **(B)** of the *motion and colour phase* for L1- and L2-regularised networks. **(C)** Influence of λ on the frequency of insight like behaviour in L1- vs L2-networks. The frequency of insight-like switches declines with increasing λ for L1-regularised networks, but is largely unaffected for L2-regularised networks. **(D)** Influence of λ on the average switch points. The average switch point occurs later in the task with increasing λ for both L1 and L2-regularised networks. Error bars signify SEM.

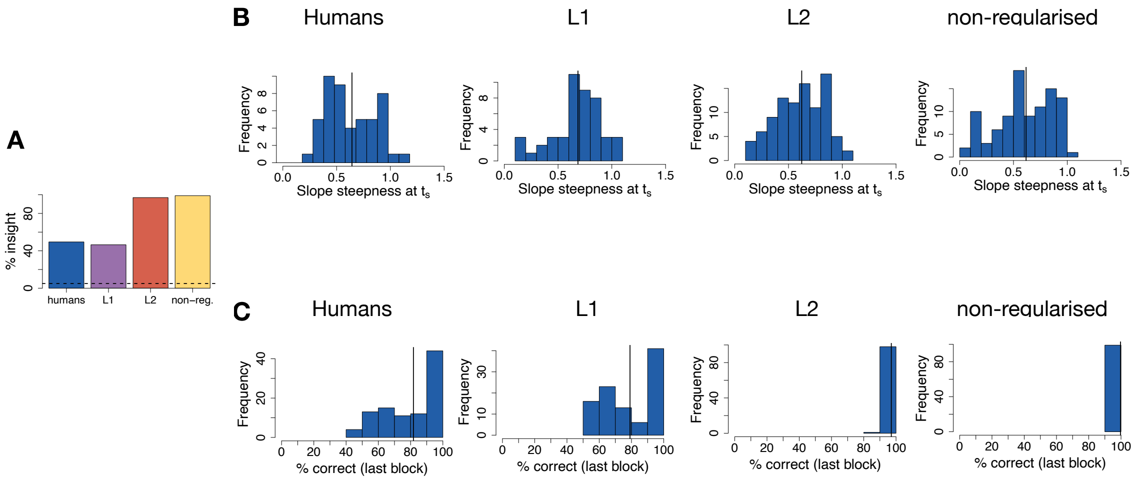

Fig J: Comparison of insight percentage and performance in the last task block across groups **(A)** % insight-like switches in humans, L1, L2 and non-regularised networks, respectively. Dashed line marks chance percentage of "insight". **(B)** Distributions of slope steepness for humans, L1, L2 and non-regularised networks. **(C)** Distributions of performance (% correct) for humans, L1, L2 and non-regularised networks for the last block of the *colour and motion phase* before the colour instruction.
