# Supplementary material for: Abrupt and spontaneous strategy switches emerge in simple regularised neural networks
Source: PLoS Comput Biol. 2024 Oct 21;20(10):e1012505. doi: 10.1371/journal.pcbi.1012505 (PMC11527165; doi:10.1371/journal.pcbi.1012505)
Supplement: Attachment — Submitted filename: Switchnet_Rebuttal.pdf [file pcbi.1012505.s002.pdf]

Dear Editor,

We are very pleased that reviewers thought favourably of our work, and would like to thank them for a thorough reading, helpful comments and suggestions.

In the revised manuscript, we have addressed all points raised by the Reviewers. In particular, we significantly improved the overall clarity of the text and discussion, clarifying our specific modelling choices, the generalisability of our proposed mechanisms for insight-like behaviour and specifically the role of regularisation.

Below we reproduce the reviewers' comments and our corresponding responses to each of them. Changes to the manuscript are also reproduced below where appropriate and highlighted in yellow in the accompanying manuscript file.

Reviewer 1

Comment 1:

However, for the human study, I have one concern which I would like the authors to address. The authors excluded almost half the participants (96 participants) from their analysis, citing insufficient accuracy levels after the motion phase. This is a very high number of subjects to exclude -- why did the authors choose the 85% threshold as the exclusion criteria?

Lastly, it would also be great to see how robust the results are when you change the threshold -- does the pattern of results still hold with different thresholds, e.g., 60% or 70%?

Response:

We thank the reviewer for raising this important point. The performance threshold of 85% for easy high coherence trials was chosen based on other experiments, in which accuracy was at a comparable level (Schuck et al., 2015; Gaschler et al., 2019; Schuck et al., 2022). A high baseline accuracy in these trials at the beginning of the task is important, since if the base strategy was not learned, an adoption of the colour strategy might reflect a different cognitive process, such as e.g. information search instead of a spontaneous strategy switch.

Unfortunately we cannot lower the threshold for this sample in hindsight, since subjects that performed below threshold (< 85% correct in low motion noise trials) were excluded from further participation, meaning we stopped data acquisition early and remunerated accordingly. We thus do not have data of the full experiment for those subjects.This also means that any post-hoc change of the exclusion criterion was not possible, alleviating any concerns about researcher degrees of freedom.
The only way we can answer your concern is more indirectly through data from a daytime nap intervention study (Loewe et al., 2024, BioRxiv), in which the same task was used and we obtained full datasets of all participants before applying the exclusion criterion. Below we show switch point aligned spontaneous strategy switches for participants classified as Insight and No insight subjects when applying an 80%, 70% or 60% performance threshold, respectively. An 80% threshold excludes 32/90 participants, 70% excludes 22/90 and 60% 17/90. As you can see,

the results are qualitatively the same. We now mention in the main text on page 20 that we chose the 85% threshold based on previous work; that a post-hoc change of the exclusion criterion was not possible, and that results from other studies indicate that the reported phenomenon is not affected by this choice:

"The 85% accuracy threshold on low motion noise trials was based on previous studies, where subjects' performance was at a comparable level [13, 16, 14]. Before starting the experiment all subjects were notified that they could only advance to the second task phase (motion and colour phase, although this was not communicated to participants) if they performed well enough in the first phase and that they would be paid accordingly for either one or two completed task phases. Based on the early stopping of the experiment for participants who performed below threshold in the motion phase, a post-hoc change of the performance criterion is not possible. Results from other studies using this or a similar task imply that the reported insight phenomenon is not an artefact of the early stopping threshold [71, 13, 15, 14]."

Threshold 80% | Threshold 70% | Threshold 60%

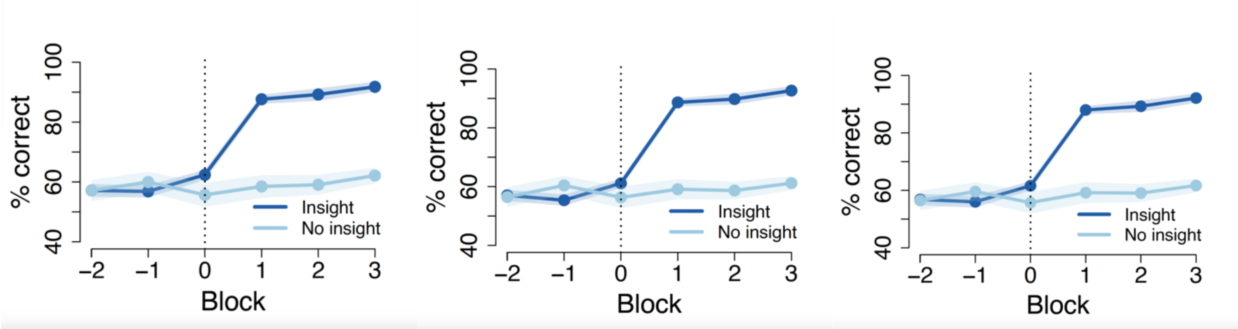

Insight (48/68) | Insight (56/78) | Insight (60/83)

No Insight (20/68) | No Insight (22/78) | No Insight (23/83)

Fig. 1: Switch-point aligned performance increases and insight ratios for performance exclusion criteria using thresholds of 80%, 70% and 60% respectively in a follow up study using the same task (Loewe et al., 2024, BioRxiv).

Reviewer 2

Comment 1:

My main critique is regarding architectural choices and their motivation in the minimal model. Most of the architecture seems standard for such basic ANNs, except for the gating mechanism. However, the motivation for this term I believe is not correspondingly spelled out. There are allusions to 'attention' and multiplicative attention is often part of complex architectures such as LSTMs or transformers, but not perceptrons. This seems to me to slightly undercut the combination of "naturally" and "simple" in the main conclusion that "insight-like behaviour can arise naturally from gradual learning in simple neural networks", at least in the sense that it would emerge in commonly used connectionist models. At the very least, the reader would want to know if the point is that attention/gating in more complex architectures is hypothesised to be the key component that enables them to (potentially) exhibit sudden insights; or alternatively is this term perhaps a simplified representation for an emergent gating mechanism in larger, but still relatively basic perceptrons?

Response:

We thank the reviewer for raising this important point. The "attention" mechanism evoked through a combination of regularisation and gating in our simple toy model is not meant to resemble the attention mechanisms in complex models such as transformers. While the gating mechanism seems to be uncommon in connectionist models, we would like to point out that our network is in fact equivalent to a two layer diagonal architecture with a linear activation function in the hidden layer, as illustrated below. The reason we chose to focus on the gating formalism is that it is easier to see the relations to non-standard neural network architectures.

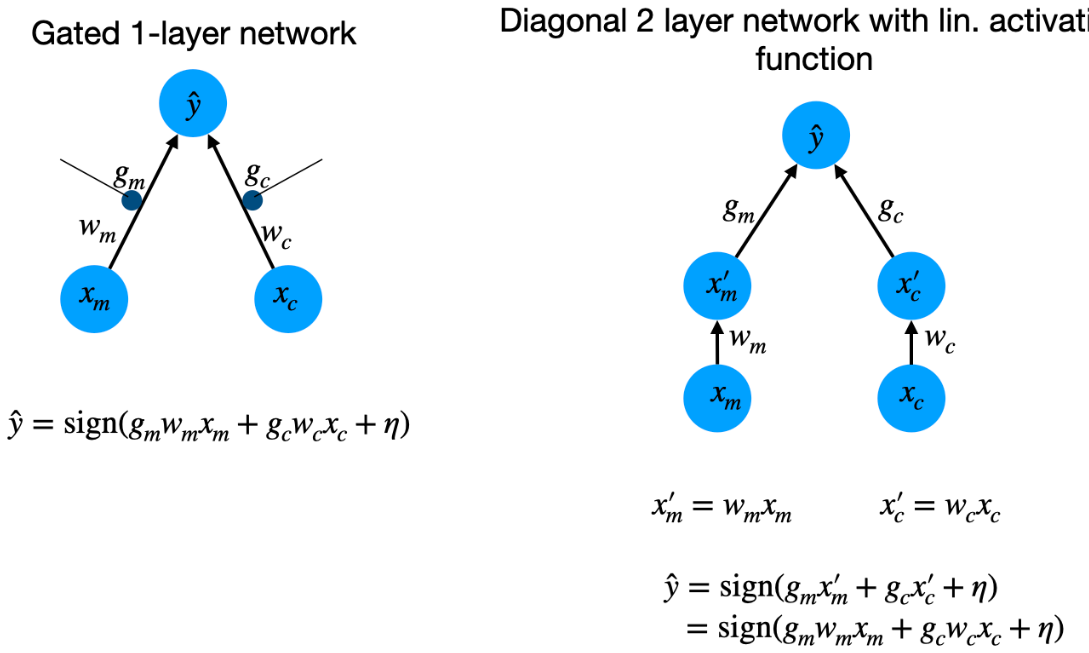

Fig. 2: Gated 1-layer network versus diagonal 2-layer network, showing the equivalence of these two architectures in our specific case.

Yet, given the equivalence we believe that our model still qualifies as a relatively simple connectionist architecture. At the same time, the gating framework makes it easier to understand that broadly, one might expect similar insight-like moments in gradient-trained models with L1 regularisation and multiplicative mechanisms — these multiplicative mechanisms could reflect depth (multiplication of layers), attention (multiplication of keys, queries, and values), gating (direct multiplication) or a combination of these. Hence we speculate that insight-like behaviour could emerge in simple models (deep networks or gated perceptrons). Whether such behaviour would emerge in more complex transformer architectures is an open empirical question. We have adapted the abstract as follows:

"*Analyses of network architectures and learning dynamics revealed that insight-like behaviour crucially depended on a regularised gating mechanism and noise added to gradient updates, which allowed the networks to accumulate ``silent knowledge'' that is initially suppressed by regularised attentional gating. This suggests that insight-like behaviour can arise naturally from gradual learning in simple neural networks, where it reflects the combined influences of noise, gating and regularisation*."

And modified the text throughout as described above.

Comment 2:

The latter possibility (emergent) seems especially relevant as sudden shifts have been shown to emerge in NNs in previous research ('rapid developmental transitions', Saxe, McClelland & Ganguli, 2018). My understanding is that the key ingredient there is the depth of the network. This citation makes an appearance in the intro, but I would advocate for also discussing how the mechanism identified here relates to the one there there, e.g., could the multiplicative gating units be seen as functionally equivalent to a hidden layer that is doing a kind of emergent gating?

Response:

The reviewer's intuition is correct - the multiplicative gating is indeed functionally equivalent to a second layer of a diagonal linear network with L1 regularisation on the second layer, see our illustration above. We therefore adopted the gating interpretation, but the same phenomena could arise in a diagonal deep linear network with regularisation at the second layer.

Fig. 10 in the Supplementary Material points towards the fact that although a standard deep linear network like the one in Saxe et al. exhibits step-like transitions, they occur reliably for all networks at a consistent latency with respect to the moment when colour becomes relevant.

However, the equivalence between the gating mechanism and a diagonal linear network is only true for the simple case that we are considering. Once it is a network with many neurons, these two accounts could be different. In order to clarify this point in the manuscript, we have now added the following statements. On page 9 directly after describing the network architecture, we write:

"*We note that our architecture is functionally equivalent to a 2-layer diagonal linear network with L1 regularisation on the second layer. Hence, although we focus on the gating interpretation, our results can equally be interpreted as pertaining to a particular simple form of linear 2-layer neural networks*"

In addition, in the discussion on page 17, we have added:

"Furthermore, since our architecture is functionally equivalent to a 2-layer diagonal linear network with L1-regularisation on the second layer, our results could equally pertain to a particular simple form of a linear network with 2 layers."

Comment 3:

Similarly, it was not clear to me why the regularisation term only applies to the gating weights g_i and not the others (w_i)?

Response:

The regularisation term is only applied to the gating weights because we hypothesised on a conceptual level that capacity constraints during the output stage act like a regularisation mechanism, while earlier processing weights should remain unaffected by this. As we described above, the regularised gate weights are equivalent to a second linear node, where the node closest to the readout would be affected by the regularisation mechanism.

Note that if both gates and weights are L1-regularised (or L2), it is impossible for the network to learn about either rule, based on motion or colour information (see below).

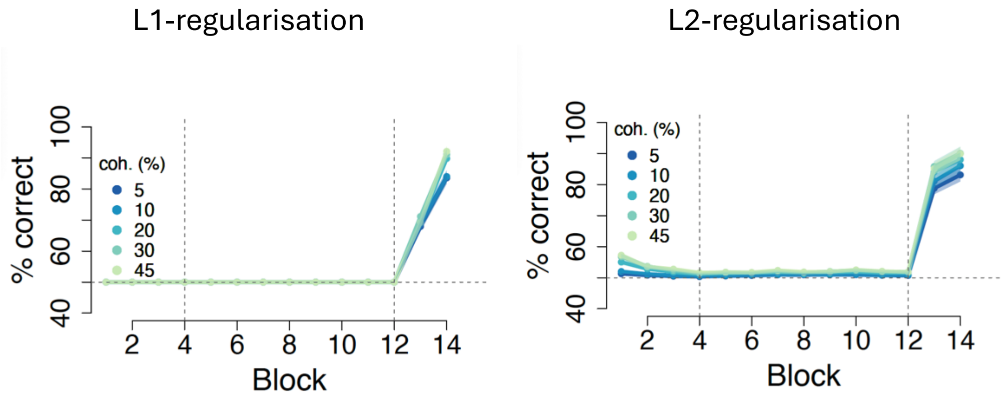

Fig. 3: L1- and L2-regularised networks with regularisation applied to gates and weights. Networks are unable to learn on the task if both gates and weights are regularised.

We note, however, that regularising only the weights, but not the gates, would have functionally equivalent effects, since this would be mathematically identical.

In the discussion on page 17, we now add the motivation and implications of our choice by writing:

"*Our choice to include explicit regularisation of only the gates was motivated by the idea that capacity constraints enforce regularisation only during the output stage, while the initial sensory processing is unaffected. Note, however, that regularising only the weights, but not the gates, would have functionally equivalent effects, and regularisation of both suppresses any learning*."

Comment 4:

In line 76: "Technically speaking, regularisation refers to adding a penalty term to the error function that prevents coefficients from reaching large values, and which thereby leads to suppression of input features [41]" I don't think this is accurate or consistent with the perspective of [41], specifically, it muddles the distinction between goal (regularisation) and mechanism (L1 and L2 penalty). Regularisation in general is a method that is intended to prevent overfitting and help with generalisation, a penalty term for large weights being one of the simplest such approaches (but there is also e.g. early stopping, data augmentation and weight sharing, all discussed in section 5.5 in the cited book [41]). In addition to the mentioned sentence, regularisation seems to be used interchangeably with the specific mechanism throughout the paper, but it is not at all made clear why that would be warranted. This is especially confusing in the discussion section where it seems like the connections are often made with regularisation in the broad sense. Do the authors hypothesise that regularisation, understood broadly, is a key component in insight-like behaviour (i.e. in a way that also translates to dreaming as is suggested in the discussion [68]), as opposed to the specific mechanism in this simple neural network happening to interact with another mechanism (multiplicative gating) that way? If yes, what supports this in the current results?

Response:

We thank the reviewer for raising an important point which refers to the distinction between explicit and implicit regularisation. It is also of course true that L1 and L2

regularisation are only two mechanisms of explicit regularisation. While implicit regularisation as a goal can be achieved with various other mechanisms as mentioned by the reviewer, these mechanisms have not been investigated in the current paper. We have hence made this distinction clear on page 3 of the introduction of the revised manuscript:

"A simple mechanism to attain regularisation involves adding a penalty term to the error function that prevents coefficients from reaching large values, and which thereby leads to suppression of input features. While these forms of explicit regularisation share similarities with other (implicit) regularisation techniques, these two forms are not identical and we focus only on the former. "

In order to avoid the confusion the reviewer points out, we are more precise throughout the manuscript in referring specifically to L1 regularisation. In the discussion, we indicate that only speculative conclusions can be drawn concerning the potential roles of other forms of regularisation, highlighting again that our current results speak for the specific combination of L1-regularisation, multiplicative gating and noise for promoting insight-like behaviour.

In the discussion on page 18, we have added:

"Previous work has also suggested the occurrence and possible usefulness of regularisation in the brain. While our simulations focused on the specific explicit regularisation mechanism of adding a penalty term to the error function, many other forms of implicit regularisation, such as weight sharing, might exist and be possibly implemented in the brain. To what extent our results generalise to such other regularisation techniques is unknown and speculative at this point. "

Comment 5:

While reading the section where the neural network model is defined, I was trying to evaluate the relative magnitudes of noise sources that are introduced (outcome, gradient, perceptual), but ran into difficulties. I couldn't find the value for σ, I might have missed where it is defined but would it be possible to mention this around the definition of Eq1.? Then I was also a bit puzzled by the stimulus

representation, for example why colour vs motion were modelled as difference in means vs variance? I see how it probably doesn't make a difference in the end but makes the relation between the experiment and the dynamics of the NN more abstract. In general, I think it would be helpful to clarify the modelling choices for the noise sources a bit, preferably not far from Eq 1-4.

Response:

We thank the reviewer for thoughtful reading of our modelling choices and have adapted the manuscript to make these clearer. In our previous version, the value for σ was defined just below Eq 2, and we have added it now above Eq 1 as well.
As for the stimulus representation, variance is kept constant for colour and motion, modelling perceptual noise, while the colour mean is constant to simulate a high stimulus to noise ratio (SNR) and motion means on the other hand model different SNR levels. These modelling choices could be switched around (constant means and different SNR variance levels) without making a difference to the results. This was added below Eq 4 on page 10:

"While σ models perceptual noise, signal to noise ratio, and therefore the difficulty, is determined by the ratio of the means M_m and M_c to sigma. Hence, different difficulty levels in the motion inputs could have equivalently been modelled as changing variances in the presence of a constant mean. "

Comment 6:

In summary, I think this is an interesting paper, however it focuses on a restricted toy model. On the one hand, this it allows for a clear, mechanistic explanation of the emergence of insight-like behaviour. On the other hand, I feel the motivation for some of the modelling choices are not made sufficiently clear, similarly to the question of how the authors propose this mechanism generalises to non-toy networks on non-toy problems. It would seem useful to me to at least gesture towards what kind of research the understanding achieved in this paper would imply regarding more realistic scale networks and be specifically on what level of abstraction the observations are thought to transferable.

Response:

We thank the reviewer for the positive remarks on the paper. As stated by the reviewer, our main goal was to pinpoint clear mechanistic insights into the computational underpinnings of insight-like strategy switches. We hope that the modelling choices have now been made more clear in the revised manuscript.

While general scalability remains an issue for future research, we do believe the mechanistic insights from our toy model have implications for more complex systems, such as the brain. To summarise our efforts in clarifying how far our results can or cannot be generalised, we highlight in the manuscript that (a) our results replicate using a multi-neuron, deep, non-linear model with a 48-unit hidden layer (paragraph “Deep Neural Networks” on page 10 and “Spontaneous Strategy Switches in Deep Neural Networks” on page 12, as well as “Hidden Layer Model” on page 2 of the Supplementary material) and (b) investigate the effects of L1 vs L2 vs no regularisation (“Effects of Regularisation Type on Network Behaviour” on page 1 and “Weight and Gate Differences between L1- and L2-regularised Networks” on page 3 of of the Supplementary material). While L2-regularised networks show abrupt behavioural transitions, they fail to show the other two key characteristics of human insight: selectivity and delay, which is emphasised even more in non-regularised networks.

We further (c) explain that our network is identical to a 2-layer diagonal network with L1-regularisation on the second layer (“Architecture” on page 9 and in the Discussion on page 17) and (d) that L1-regularisation of weights has equivalent effects of gate regularisation, while L1-regularising both entirely changes the model behaviour.

We speculate that even more complex, deeper networks, which would allow to disentangle several factors of variation (e.g. motion, shape, colour, lighting) and apply gating variables to these, might also exhibit similar shifts between relying on subsets of these features. The most specific implication for research is the suggestion of a link between measurements of noise, and regularisation in the brain, and behavioural markers of insight.

To emphasise this in the paper, we added this paragraph to page 19 of the discussion:

> “*While general scalability remains an issue for future research, we believe that the mechanistic insights from our model have implications for more complex systems, such as the brain. Our results imply a link between behavioural markers of insight and noise measurements as well as regularisation in the brain. Towards this goal of generalisation we replicated our results using a multi-neuron, deep, non-linear model with a 48-unit hidden layer and investigated effects of different types of explicit regularisation (L1 vs L2 vs no regularisation), showing that only L1-regularised networks exhibit all three key characteristics of human insight. We further explained that our network is identical to a 2-layer diagonal network with L1-regularisation on the second layer and that L1-regularisation of weights has equivalent effects of gate regularisation, while L1-regularising both entirely changes the model behaviour. We speculate that even more complex, deeper networks, which would allow to disentangle several factors of variation (e.g. motion, shape, colour, lighting) and apply gating variables to these, might exhibit similar shifts between relying on subsets of these features.*”

> As a side note, the reviewer might be interested to hear that a follow up study to this experiment that investigated the effect of a daytime nap intervention on insight (Löwe et al., 2024, BioRxiv), found that the steepness of the spectral slope during sleep, which has been indirectly linked to regularisation (Lendner et al., 2023, Science Advances), predicted insight above and beyond sleep stages. The relationship between insight-like behaviour and regularisation in the broader sense - and different regularisation mechanisms specifically - thus remains an interesting area for future research.

We have added this to the discussion on page 19:

> “*Since our experiments were conduced in an uninterrupted fashion during daylight, our findings could not reflect any sleep effects. The findings above nevertheless suggests a possible link between sleep, synaptic scaling and insight. A follow up study to this experiment that investigated the effect of a daytime nap intervention on insight (Löwe et al., 2024, BioRxiv), found that the steepness of the spectral slope during sleep, which has been indirectly linked to regularisation (Lendner et al., 2023, Science Advances), predicted insight above and beyond sleep stages. The relationship between insight-like behaviour and regularisation in the broader sense -*

and different regularisation mechanisms specifically - thus remains an interesting area for future research. ”

We are now more clear about this perspective by stating in the abstract:

“*These results have potential implications for more complex systems, such as the brain, and guide the way for future insight research.*”

In the discussion, we generally discuss these issues at length on pages 18 and 19, where we state, amongst others:

“*Albeit our conceptual model was not meant to be biologically realistic, this work adds a computationally precise explanation of how noise can lead to insight-like behaviour to this literature. Questions about whether inter-individual differences in neural variability predict insights (Garrett et al., 2013), or about whether noise that occurs during synaptic updating is crucial remain an interesting topic for future research.* ”

Minor comments:

Comment 7:

Figure 1E, y axis is only made clear in main text, would be good to point it out either on figure or in caption

Response:

This has been addressed in the updated manuscript.

Comment 8:

Fig 1 caption says "classification of insights versus no insight agreed to 79.6% with verbal insight reports" then in the main text "Of the 49 participants classified as insight subjects 39 (79.6%) also self-reported to have used colour to make correct choices". This doesn't seem to be what I'd understand as agreement between two

classifiers, I'd assume one would also want to take into account how much the responses of non-insight subjects (according to self-report) agree with the behavioural measure, maybe this is just unclear phrasing?

Response:

The self-reported non-insight subjects that were classified as insight participants make up the remaining 20.4% (10/49). We have clarified this in the revised manuscript.

Comment 9:

line 271, "in and off"->in and of

Response:

This has been addressed in the updated manuscript.
